# Antineoplastic effects of targeting CCR5 and its therapeutic potential for colorectal cancer liver metastasis

**DOI:** 10.1007/s00432-020-03382-9

**Published:** 2020-09-09

**Authors:** Asim Pervaiz, Michael Zepp, Rania Georges, Frank Bergmann, Saqib Mahmood, Syeda Faiza, Martin R. Berger, Hassan Adwan

**Affiliations:** 1 Institute of Biomedical and Allied Health Sciences, University of Health Sciences, Lahore, Pakistan; 2 Toxicology and Chemotherapy Unit, German Cancer Research Centre (DKFZ), Heidelberg, Germany; 3 Institute of Pathology, University of Heidelberg, Heidelberg, Germany; 4 German University of Cairo, Cairo, Egypt

**Keywords:** Colorectal cancer, Liver metastasis, CCR5 inhibition, Maraviroc, Anticancer effects, Therapeutic target

## Abstract

**Purpose:**

Liver metastasis is observed in up to 50% of colorectal cancer (CRC) patients. Available treatment options are limited and disease recurrence is often. Chemokine receptor 5 (CCR5) has attracted attention as novel therapeutic target for treating cancers. In this study, we reinforced the importance of CCR5 as therapeutic target in CRC and its liver metastasis by applying in vitro, in vivo and clinical investigations.

**Methods:**

By targeting CCR5 via siRNAs or an FDA approved antagonist (maraviroc), we investigated the ensuing antineoplastic effects in three CRC cell lines. An animal model for CRC liver metastasis was used to evaluate time-dependent expressional modulation of the CCR5 axis by cDNA microarray. The model was also used to evaluate the in vivo efficacy of targeting CCR5 by maraviroc. Circulatory and tumor associated levels of CCR5 and its cognate ligands (CCL3, CCL4, CCL5) were analyzed by ELISA, qRT-PCR and immunohistochemistry.

**Results:**

Targeting the CCR5 inhibited proliferative, migratory and clonogenic properties and interfered with cell cycle-related signaling cascades. In vivo findings showed significant induction of the CCR5 axis during the early liver colonization phase. Treatment with maraviroc significantly inhibited CRC liver metastasis in the animal model. Differential expression profiles of circulatory and tumor associated CCR5/ligands were observed in CRC patients and healthy controls.

**Conclusion:**

The findings indicate that targeting the CCR5 axis can be an effective strategy for treating CRC liver metastasis.

**Electronic supplementary material:**

The online version of this article (10.1007/s00432-020-03382-9) contains supplementary material, which is available to authorized users.

## Introduction

CRC is the 3rd leading cause of cancer-related mortalities worldwide (Ferlay et al. 2015; Siegel et al. 2016). CRC is a highly malignant disease with tumor cells having a tremendous ability of metastasizing to distant organs, including liver, lungs, bones and brain. Among these potential organs, liver is the pre-dominant site for CRC metastasis, as it offers both, a suited soil and the first vascular bed for the circulating tumor cells (Sheth and Clary 2005; Valderrama-Trevino et al. 2017; Zarour et al. 2017). At the first round of medical check-up/ surgery for primary CRC, almost 15–20% of the patients are diagnosed with liver metastasis (synchronous metastasis). Furthermore, a significant proportion of CRC patients (> 50%) develop liver metastasis over the course of the disease (metachronous metastasis) and account for a major fraction of CRC associated mortality (Helling and Martin 2014; Jegatheeswaran et al. 2013; Valderrama-Trevino et al. 2017). Hepatic resection, along with systemic adjuvant regimens, is the present day therapeutic option for CRC liver metastasis, but it cures only a limited proportion of the patients (< 20%) and often cannot inhibit disease recurrence (House et al. 2011; Konopke et al. 2012; Tol and Punt 2006; Tomlinson et al. 2007). To summarize, after liver metastases have been established, available surgical and combinational therapies are of minor assistance to cure the disease, leading to a significantly reduced 5-year survival rate (10–15%) (Adam 2007; Adam et al. 2015; Alberts 2012; Riihimaki et al. 2016). In this scenario, it is of paramount importance to identify new therapeutic targets and means for improving the treatment options to possibly cure CRC liver metastasis.

In recent years, the chemokine network has been exploited extensively in search of new prognostic markers and therapeutic targets for treating cancers. Chemokines are basically a class of secretory chemo-attractant cytokines (8–14 kDa), which mediate a variety of physiological functions including cellular migration, development, survival, inflammatory responses and angiogenesis (Hughes and Nibbs 2018; Raman et al. 2011). In addition to their homeostatic and inflammatory functions, chemokines are being investigated for their potential role in cancer progression. Multiple aspects of tumor biology like angiogenesis, leukocyte infiltration and metastasis are affected via the chemokine network in an auto- and/or paracrine manner (Lacalle et al. 2017; Liu et al. 2017; Lopez-Cotarelo et al. 2017; Massara et al. 2016). In view of their significant pro- or anti-cancer effects, strategies are being developed to exploit the chemokine network for therapeutic purposes. Along these lines, the developed entities for targeting the chemokine network including antibodies, antagonists or small molecules are being investigated in pre-clinical settings or even clinical trials (Mollica Poeta et al. 2019; Mukaida et al. 2014).

Alterations in chemokine expression levels have been witnessed during CRC development, invasion and metastasis. Subsequent effects of these modulations mainly depend upon the type of chemokine, the corresponding concentration and on source/target cells (Emmanouil et al. 2018; Itatani et al. 2016; Ryu et al. 2018). CCR5 (CD195) along with its three known ligands (CCL3, CCL4, CCL5) comprises an important axis of the chemokine network and mediates multiple physiological functions as well as others related to malignancies including CRC (Aldinucci and Casagrande 2018; Fuente et al. 2018; Oliveira et al. 2014; Singh et al. 2018; Walens et al. 2019). These multifunctional properties of CCR5 are largely attributed to the expression of this receptor on a variety of cells including leukocytes, stromal and cancer cells. A differential expression profile of the CCR5 axis has been reported in CRC and its liver metastasis. As far as the functional importance of CCR5 is considered, the majority of the reports have supported a pro-tumor role of the CCR5 axis in CRC progression (Chang et al. 2012; Pervaiz et al. 2015; Sasaki et al. 2014; Schimanski et al. 2011). Owing to its vital role in CRC progression, the CCR5 axis is currently in the spotlight of consideration as a therapeutic target. Numerous strategies including development of specific antagonists and antibodies are being deployed to block the CCR5 axis of CRC cells for therapeutic purposes. In addition, CCR5 blockage on other cells including cells of the immune system has been proposed to be very effective in reducing CRC burden and its liver metastasis (Halama et al. 2016; Tanabe et al. 2016). Nevertheless, development of more specific and clinically relevant CCR5 inhibitors to target this chemokine axis in cancers is a continuing process.

In this study, following the CCR5 inhibition using gene specific siRNAs or an FDA approved antagonist (maraviroc), we investigated the role of CCR5 receptor in CRC progression and metastasis in pre-clinical settings. For this purpose, human (SW480, SW620) and rat (CC531) CRC cell lines were used in a series of in vitro assays. Furthermore, time-dependent modulations in expressional profiling of the CCR5 axis during CRC liver metastasis were determined using a related animal model. In addition, the potential of CCR5 inhibition by maraviroc was assessed regarding its capability to restrict CRC liver metastasis progression in vivo. Furthermore, we measured the circulatory and tumor associated levels of CCR5 and its ligands (CCL3, CCL4, CCL5) in serum (ELISA), primary tumors (qRT-PCR; IHC) and matched liver metastases (IHC) from CRC patients to assess potential morbidity-related changes.

## Materials and methods

### Cell lines and chemicals

Human (SW480 and SW620) and rat (CC531) colon adenocarcinoma cell lines were obtained from American Type Culture Collection (ATCC, Manassas, VA, USA) and Cell Line Service (CLS, Eppelheim, Germany), respectively. The cells were cultured and maintained under standard incubation conditions (5% CO2, 37 ˚C, humidified atmosphere) in Roswell Park Memorial Institute medium (RPMI-1640) supplemented with 2 mM L-glutamine, 10% fetal bovine serum (FBS, Gibco: 10,270–106), 100 µg/ml streptomycin and 100 IU/ml penicillin. Cell lines were routinely tested (every 3 months) for mycoplasma contamination using the VenorGem PCR kit (Minerva Biolabs, Berlin, Germany) and passaged two to three times per week to maintain logarithmically growing cell populations. For propagation, the cells were washed with PBS, trypsinized (0.05% trypsin) and cell pellets were collected by centrifugation at 1500–2000 rpm for 5 min. The cells were counted by a Neubauer chamber and re-suspended at desired cell densities according to the experimental needs. Purified compound and commercially available tablets of maraviroc were purchased from Selleck Chemical Co. China (UK-427857) and Viiv Healthcare GmbH, Germany, respectively.

### CCR5 expression and knockdown

CCR5 expression was assessed in untreated human CRC cell lines (SW480 and SW620) using quantitative real-time PCR (qRT-PCR) and western blot methodologies as described in below sections. Afterwards, small interfering RNA (siRNA) duplexes were designed against the human CCR5 gene (Sequence 1: 5′-AUUGAUACUGACUGUAUGG-3′, Sequence 2: 5′-AGAUGAACACCAGUGAGUAGAGCGG-3′, Invitrogen), while nonspecific siRNA (mock) was purchased from Ambion, Berlin, Germany (cat#AM4615). Following the manufacturer’s instructions of the transfecting reagent (X-tremeGENE 9, Roche, Mannheim, Germany), the cells were cultured to 50–60% confluence prior to transfection with siRNAs (200 nM, 24–72 h) in 96, 24, 12, 6-well plates or 25 cm^2^ cell culture flasks as per demand of the experiments.

### Quantitative polymerase chain reaction (qRT-PCR)

CCR5 knockdown efficacy was evaluated by qRT-PCR, where total RNA was extracted from the cell pellets by using RNeasy Mini kit (Qiagen, Hilden, Germany) followed by synthesis of complementary DNA (cDNA) by Maxima reverse transcriptase (Thermo Scientific, Schwerte, Germany). The CCR5 transcript was detected using a mixture of gene specific primers (Table 1), 2X LC480 Master Mix (Roche, Mannheim, Germany) and an appropriate probe from the Human Universal Probe Library (Roche, Mannheim, Germany), which was amplified in a LightCycler 480 Real-Time PCR system. The samples were processed in triplicate and the expression level of the glyceraldehyde 3-phosphate dehydrogenase (GAPDH) gene was used as reference to normalize the data.

**Table 1:** Primer sequences

Gene symbol	Forward primer (5′ → 3′)	Reverse primer (3′ → 5′)
CCR5	AACCAGGCGAGAGACTTGTG	GATCCAACTCAAATTCCTTCTCA
CCL3	CAGAATCATGCAGGTCTCCAC	GCGTGTCAGCAGCAAGTG
CCL4	CTTCCTCGCAACTTTGTGGT	CAGCACAGACTTGCTTGCTT
CCL5	TGCCCACATCAAGGAGTATTT	TTTCGGGTGACAAAGACGA
GAPDH	AGCCACATCGCTCAGACAC	GCCCAATACGACCAAATCC

### Immunoblotting

Western blot analysis was used to assess knockdown of CCR5 at protein levels. To that purpose, the experimental cells were harvested, washed with PBS and stored in liquid nitrogen. Subsequently, to extract the protein content, the pellets were lysed with RIPA buffer (150 mM sodium chloride, 1.0% NP-40, 0.5% sodium deoxycholate, 0.1% sodium dodecyl sulfate, 50 mM Tris, pH 8.0) supplemented with complete protease inhibitor cocktail tablets (Roche, Mannheim, Germany). Afterwards, the supernatant was collected by centrifugation (14,000 rpm/4 °C, 20 min) and quantified for protein concentration using the Pierce Protein Assay. The total protein lysates (30–50 µg) were subjected to electrophoresis on 4–12% gradient polyacrylamide SDS gels followed by transfer onto PVDF membranes and probing for CCR5 protein using specific primary antibody as per manufacturer’s instructions (Cell Signaling Technologies, Frankfurt, Germany). Immunoblots were developed using a HRP-conjugated anti-mouse (Cell Signaling Technologies, Frankfurt, Germany) and ECL-System (Amersham Pharmacia Biotech, Munich, Germany). Levels of β-actin (Santa Cruz Biotechnology) were used to normalize the data, and relative concentrations were measured by densitometric analysis of digitized autographic images using the ImageJ Program.

### Cell proliferation assay

Cell proliferation was assessed by MTT (3-[4,5-dimethylthiazol-2-yl]-2,5 diphenyltetrazolium bromide) dye reduction assay. In brief, the cells were counted in a Neubauer’s chamber, suspended in RPMI-1640 complete medium and seeded in 96-well plates at pre-optimized cell density (5 × 10^3^ cells/100 μl medium/well). After an incubation period of 24 h, CCR5 was knocked-down by siRNA or blocked by increasing concentrations of purified maraviroc (1.5–750 µM) dissolved in ethanol (100 mM stock). Correspondingly, highest ethanol (the vehicle) concentrations used were ≤ 0.75% (by volume) in any of the samples exposed to maraviroc. Following treatment, the cells were incubated at standard conditions for 24, 48 or 72 h. Thereafter, MTT solution (10 mg/ml in PBS) was added (10 μl/well) and plates were incubated for another 3 h in the incubator. Afterwards, the old medium was discarded and formed crystals of formazan were dissolved by adding 100 μl/well of acidified solvent (0.04 N HCl in 2-propanol). Optical densities were measured by an ELISA plate reader at 540 nm absorbance wavelength and 690 nm reference filters. Cell survival rates were shown as percentage of controls transfected with mock siRNAs, or treated with equal concentrations of the vehicle (ethanol), while the inhibitory concentrations (IC) were calculated by GraphPad Prism 6 software.

### Colony formation assay

The effects of targeting CCR5 on clonogenic ability of CRC cells were assessed by colony formation assay. Following siRNA-mediated knockdown of CCR5 or blockage by maraviroc IC_20_ (SW480: 213 µM, SW620: 148 µM, CC531: 432 µM) for 48 h, 5 × 10^2^ cells/1.5 ml semiliquid medium (0.4% methylcellulose and 30% FBS in RPMI-1640 medium) were transferred to six-well plates. After an incubation period of 6–8 days under standard culture conditions, clusters of cells were counted by an inverted microscope (Leitz Fluovert FU Microscope, Wetzlar, Germany). Clusters with more than 10 cells were recorded as colony-forming units and categorized as small (< 30 cells) or large (≥ 30 cells) colonies. Data sets were represented as percentage of the controls (mock transfected siRNA or treated with the vehicle only).

### Migration assay

To study the effects of targeting CCR5 (siRNA/maraviroc) on directional migration of the cells, we used a two compartment model separated by an 8 μm polycarbonate membrane (Millicell, Millipore, Germany) with a chemo-attractant (FBS) in the lower compartment. Briefly, the bottom of 24-well plates was covered with 250 μl of FBS, then gently over layered by 650 μl semi-liquid medium (0.4% methylcellulose and 20% FBS in RPMI-1640 medium) and incubated under standard conditions for 24 h to build a chemotaxis gradient. Following the transfection with CCR5 or mock siRNAs for 48 h, the cells were counted and equal numbers were seeded (5 × 10^4^ cells/200 ul Optimem media) into hanging Millicell inserts with polycarbonate membrane. For the maraviroc group, respective cell numbers were transferred to Millicell inserts and allowed to migrate in the presence of compound (IC_20_) or vehicle only. Migrating cells were counted under an inverted microscope (Leitz Fluovert FU Microscope, Wetzlar, Germany) for 24, 48, and 72 h time intervals, while the filters with non-migrated cells were placed each day onto wells of a new plate with fresh chemotaxis gradient.

### Wound healing assay

The effect of targeting CCR5 on CRC cell mobility was assessed by using a wound healing assay. In brief, the cells were seeded in 12-well plates (1 × 10^5^ cells/well) and allowed to grow as monolayer under standard incubation conditions. Next day, the cells were knocked-down with respective siRNAs (gene specific or mock) for 48 h, followed by the creation of a straight scratch using a 200 μl sterile pipette tip. Free-floating cells from the wells were removed carefully and optimum medium (500 µl/well) with reduced FBS (0.5%) was added. With regard to the CCR5 antagonist (maraviroc), the cells were exposed to the compound (IC_20_) or vehicle only after 48 h of seeding. The images were captured by Axio Observer Z1 microscope (Carl Zeiss, Oberkochen, Germany) in both cases for zero and 24 h to monitor the “scratch healing” process.

### Cell cycle panel and signaling pathway

In a previous study, we reported that targeting CCR5 by maraviroc induces significant arrest in G0/G1 phase of cell cycle in CRC cells (Pervaiz et al. 2015). To figure out the mechanistic reasoning for these previously observed effects, we used a ready-made Human Cell Cycle Regulation Panel (Cat. 05,339,359,001, Roche) and qRT-PCR methodology. The panel contains probes/primers for 84 cell cycle relevant genes (Supplementary File 1), appropriate controls (genomic DNA, RT-negative and positive controls) and 7 reference genes to monitor overall amplification and normalization of the data. Briefly, metastatic CRC cells (SW620) were exposed to maraviroc (IC_75_/48 h) followed by extraction of total RNA and cDNA synthesis as described above. qRT-PCR was performed using 50 µl cDNA (0.5 µl/well) prepared from 1000 ng extracted RNA along with 2X LC480 Master Mix (Roche, Mannheim, Germany) in a LightCycler 480 Real-Time PCR System. After normalization of the data sets, relative fold changes were calculated by the 2− △△Ct method. Based on the results from this panel, a signaling pathway was predicted with the help of *Ingenuity Pathway Analysis* software (Redwood, USA) at the Proteomics and Genomics core facility of DKFZ, Heidelberg.

### Microarray analysis

Microarray analysis was performed to highlight the expressional modification in CCR5 and its cognate ligands (CCL3, CCL4, and CCL5) during the process of CRC liver metastasis (Georges et al. 2012). In brief, RFP-labelled CRC cells (CC531) were transplanted to the rat liver via the hepatic portal vein, which creates an animal model mimicking liver metastasis. Transplanted cells were re-isolated by FACS after discrete time intervals (3, 6, 9, 14 and 21 days) followed by RNA extraction with the RNeasy Mini kit (Qiagen, Hilden, Germany). In addition to this, a fraction of re-isolated cells was cultured in vitro for 14 and 22 days to compare the results with those from tumor cells grown in vivo. Following the extraction procedure, quality of extracted RNA was determined by gel analysis while using the total RNA Nano chip assay on an Agilent 2100 Bioanalyzer (Agilent Technologies GmbH, Berlin, Germany). RNA samples with RNA integrity number (RIN) values ≥ 8.5 were selected for further expression profiling.

## In vivo studies

### Animal husbandry

Male WAG/Rij rats (6–8 weeks old/ 150–175 g weight) were purchased from Charles River (Sulzfeld, Germany), fed with standard diet (ad libitum) and kept under specific pathogen free (SPF) controlled conditions (22 °C ± 1 °C temperature, 55% humidity and 12 h dark/light rhythm).

### Implantation of CRC cells and treatment

The rats, after an adaption period of 1 week, were implanted with a single cell suspension of CC531 cells (4 × 10^6^ cells/rat) transfected with marker genes (eGFP/RFP/luciferase) as described previously (Georges et al. 2011). Following the implantation procedure, the rats were divided randomly into three groups; A: control (eight rats/group), B: gemcitabine (four rats/group) and C: maraviroc (six rats/group). Treatment of group C was started from 2nd day of transplantation with maraviroc extracted from commercially available tablets using 100% ethanol (50 mg/ml stock). For treatment purposes, a mixture of maraviroc (25 mg/kg/rat), KolliphorR EL (cremophor EL) as emulsifier (100 µl/rat) and double distilled autoclaved water was prepared (500 µl/rat). Treatment was continued on a daily basis for 3 weeks by intra-peritoneal delivery of the compound. For group B, treatment was started from day 2 of implantation with gemcitabine (50 mg/kg/rat, intra-peritoneal delivery) once per week and carried out for 3 weeks. The control group was treated with a mixture of ethanol/cremophor EL/water (vehicle) for the same period.

### Treatment response follow-up

Tumor growth was assessed in treated and control groups of rats by monitoring the luciferase activity of transfected CRC cells. For this purpose, the rats were injected with a solution (500 µl/rat) of sodium-D-Luciferin (5 mg/rat) in PBS. Light emission signals resulting from the luciferase-mediated metabolism of luciferin were recorded by the IVIS 100 imaging system (Xenogen Corp., California, USA) for a total period of 3 weeks. At the end of experiments, all rats were sacrificed and livers were excised carefully, washed with PBS and weighed.

## Clinical investigations

### ELISA

Sera were separated using serum separator tubes from the blood samples of 24 naïve CRC patients. Following the sera separation, circulatory levels of the three CCR5 receptor cognate ligands (CCL3, CCL4, CCL5) were measured by enzyme-linked immunosorbent assay (ELISA) using the single-analyte ELISArray kits (CCL3/SEH00566A, CCL4/SEH00563A, CCL5/SEH00703A, Qiagen, Hilden, Germany). The results were compared with those from 24 volunteer age/sex-matched healthy controls.

### Real-time PCR

Total RNA from 51 frozen CRC tissue and 10 normal mucosa specimens was isolated using the RNeasy Mini kit (Qiagen, Hilden, Germany) followed by RNA quantification by a GeneQuant Pro spectrophotometer (GE Healthcare, Munich, Germany) and cDNA synthesis (1000 ng RNA) using Maxima reverse transcriptase enzyme (Thermo Scientific, Schwerte, Germany). Prepared cDNA samples were subjected to real-time PCR for expressional analysis of CCR5 and its cognate ligands (CCL3, CCL4, CCL5). Gene specific primers (Table 1), 2 × LC480 Master Mix (Roche, Mannheim, Germany) along with appropriate probes from the Human Universal Probe Library (Roche, Mannheim, Germany) and a LightCycler 480 Real-Time PCR System were used for the amplification procedures. After normalizing the data sets, relative fold changes in transcripts were calculated by the 2-△△Ct method.

### Immunohistochemistry

Formalin-fixed and paraffin-embedded tissue sections (4 μm thickness) were obtained from 15 CRC and matched liver metastases and subjected to immunostaining as described earlier (Georges et al. 2012). Briefly, tissue sections were de-paraffinized in xylene followed by rehydration in decreasing concentrations of ethanol and washing steps with Tris-buffered saline pH 7.4 (10 mM Tris–HCl, 0.85% NaCl and 0.1% bovine serum albumin) followed by antigen retrieval by boiling of the sections in 10 mM citrate buffer for 10 min. The sections were incubated and stained with the same CCR5 antibody that was used for western blot, as per manufacturer’s instructions.

### Statistical analysis

GraphPad Prism 6 software was used for the statistical analysis of data. Student *t*-test and one-way ANOVA were used for comparison between two or more groups, respectively. Data were expressed as mean ± SD and a *P* value < 0.05 was considered statistically significant (*P < 0.05, ** *P* < 0.01, *** *P* < 0.001).

## Results

### CCR5 inhibition halts proliferation of CRC cells

**Fig. 1:**
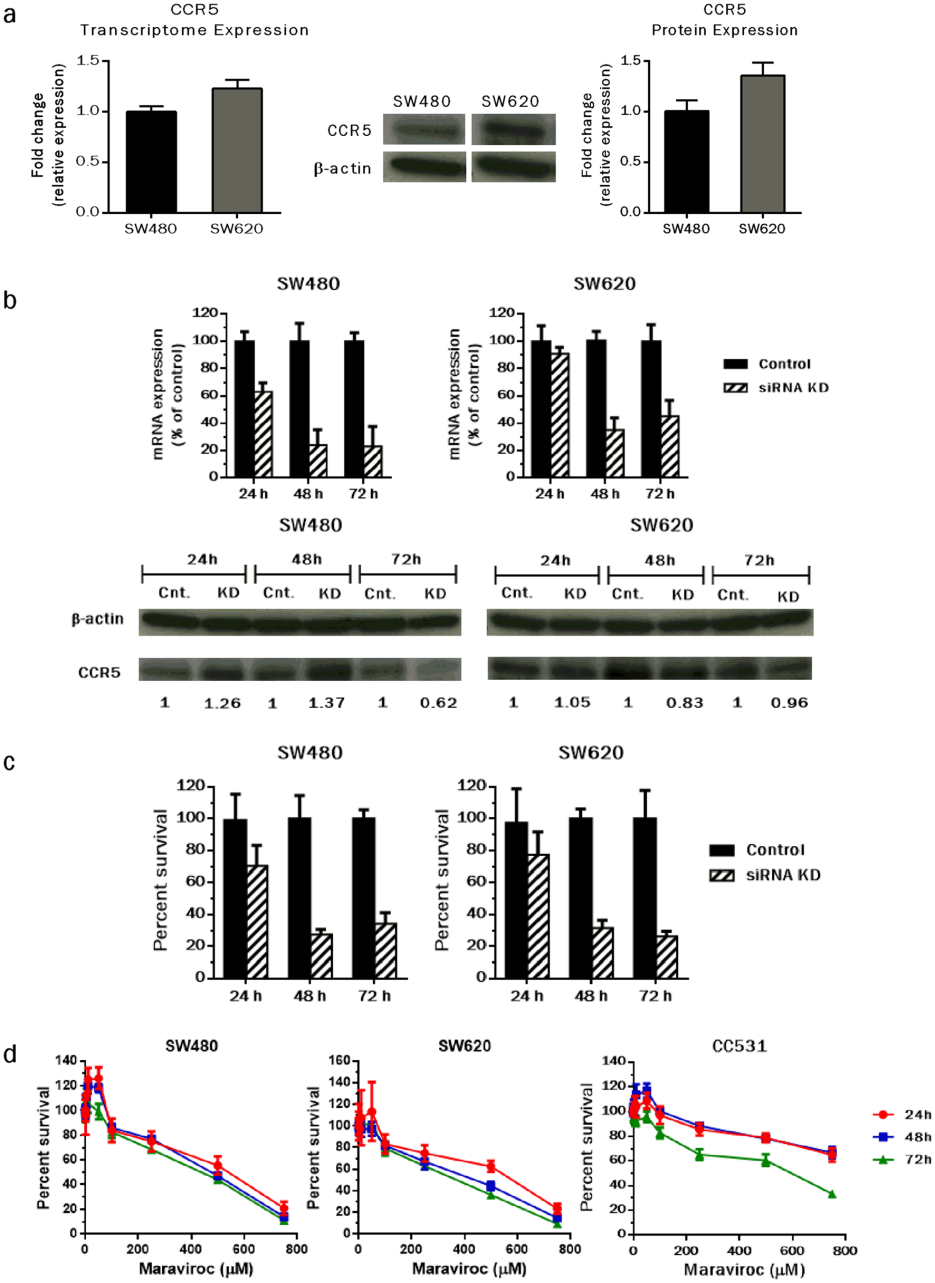
CCR5 inhibition and ensuing effects on cellular proliferation. Expression levels of the CCR5 were identified in CRC cells by qRT-PCR and western blotting. The expression was 1.22 and 1.36-fold higher in SW620 cells than in SW480 cells at mRNA and protein levels, respectively (**a**). To inhibit CCR5 expression, human CRC cells (SW480 and SW620) were seeded in six-well cell culture plates, allowed to grow overnight and transfected with gene-specific or mock siRNA using X-tremeGENE 9 as transfection agent. Knock-down efficiency was evaluated by qRT-PCR and western blot methodologies (**b**). Anti-proliferative effects of CCR5 inhibition by using gene specific siRNA or FDA approved antagonist (maraviroc) were assessed in human (SW480, SW620) and rat (CC531) CRC cells by MTT dye reduction assay. The cells were seeded in 96-well cell culture plates, allowed to grow overnight and transfected with gene specific siRNA or exposed to increasing concentrations of maraviroc (1.5–750 µM) for 24, 48 and 72 h. Bar and line graphs indicate inhibition of cell proliferation in response to CCR5 inhibition using siRNA or maraviroc, respectively (**c**, **d**). Inhibitory concentrations (IC) of the antagonist were calculated by using GraphPad Prism 6 software

Oncogenic transformation often leads to continuous cellular proliferation, a typical hallmark of cancer growth. As an initial step, the expression of CCR5 was determined at mRNA and protein levels in SW480 and SW620 CRC cells. Although, CCR5 was detectable in both cell lines, a relatively higher expression was observed in metastatic CRC SW620 cells (Fig. 1a). To evaluate the effects of CCR5 inhibition on cell proliferation, CRC cells were transfected with gene specific siRNAs or exposed to the antagonist (maraviroc). qRT-PCR demonstrated significant inhibition of CCR5 with siRNA sequence 1 (data for siRNA sequence 2 are not shown) when compared to the cells transfected with mock siRNA. The CCR5 expression inhibition was more pronounced at later time intervals following transfection (48 and 72 h). The knockdown was less effective at protein level, however (Fig. 1b). Following siRNA-mediated CCR5 inhibition or exposure to increasing concentrations of maraviroc (1.5–750 µM), the effects on cell proliferation was assessed by MTT assay. Anti-proliferative effects of maraviroc exposure on human CRC cell lines were documented in a previous study (Pervaiz et al. 2015) and reconfirmed here before proceeding to the subsequent experiments. Significant anti-proliferative effects were manifested in response to CCR5 inhibition/blockage in the human and rat CRC cells. Following siRNA-mediated CCR5 inhibition, the cytotoxic effects were almost comparable and time-dependent in selected human (SW480 and SW620) CRC cells (Fig. 1c). Remarkably, the observed effects were in line with siRNA-mediated inhibition of CCR5 mRNA expression. CCR5 blockage by maraviroc exposure also induced a steep decline in the percentage of viable cells in human and rat CRC cells in a dose-dependent format beyond 50 µM concentrations of the test compound (Fig. 1d). In contrast to the delayed siRNA-mediated anti-proliferative effects, maraviroc exposure significantly inhibited cell viability in a concentration-dependent mode starting from early time intervals (24 h). The early anti-proliferative effects of maraviroc probably result from the fact that the antagonist occupied the CCR5 receptor structures effectively upon exposure (receptor saturation), interrupted the interactions with the cognate ligands and thus led immediately to the reduced proliferation. In contrast to this, siRNA-mediated inhibition of CCR5 led to reduced expression of the receptor only after the present CCR5 receptors had been degraded and thus caused a delayed anti-proliferative effect. Furthermore, the effects were comparable in human CRC cells (SW480, IC_50_/72 h: 451 µM, SW620, IC_50_/72 h: 392 µM), while rat cancer cells were less responsive towards maraviroc exposure (CC531, IC_50_/72 h: 586 µM). Collectively, the data suggest that CCR5 inhibition/blockage halts the proliferation of CRC cells in vitro.

### Targeting CCR5 abrogates colony formation and migration of CRC cells

**Fig. 2:**
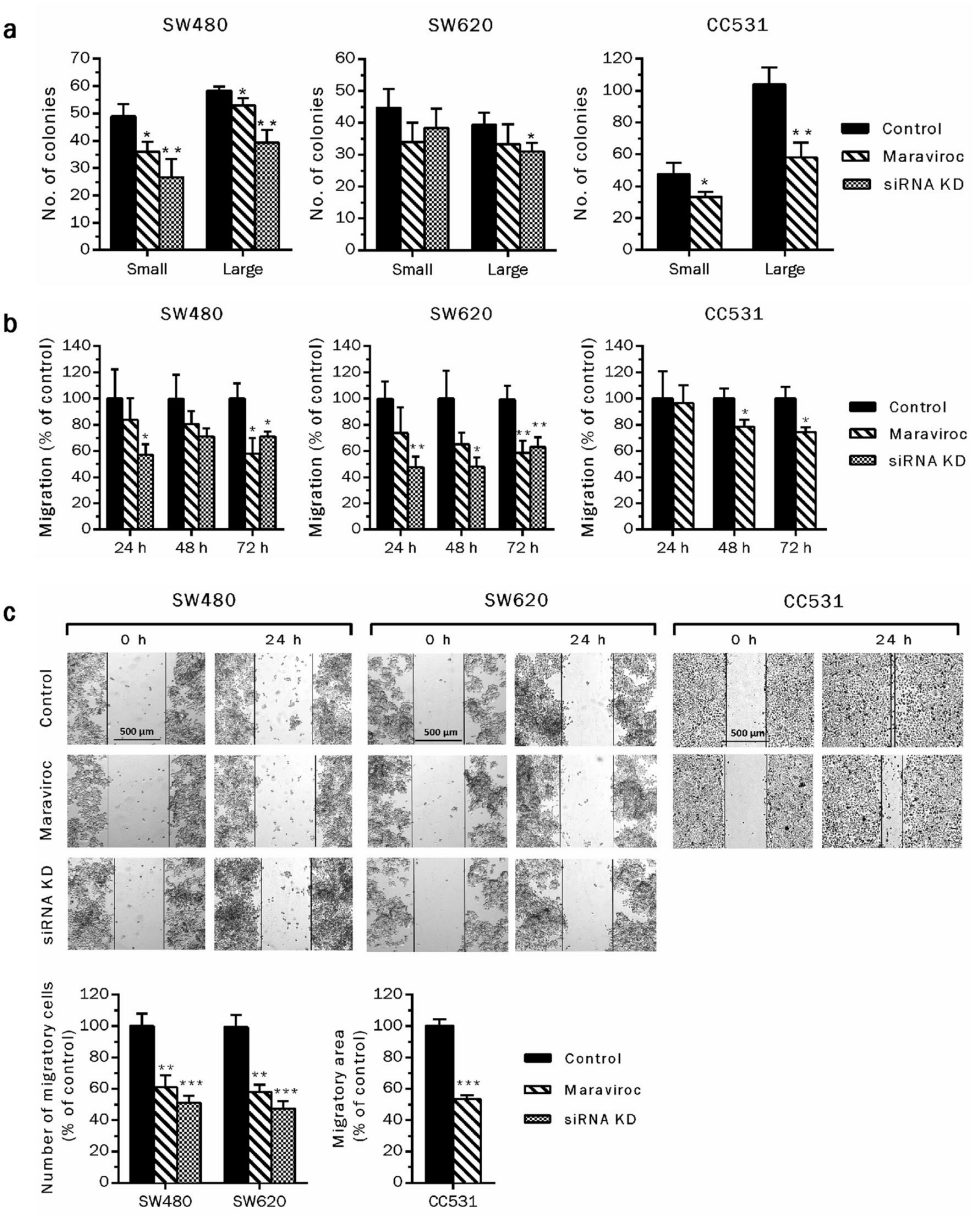
CCR5 inhibition reduces colonization and migration of CRC cells. Effects of CCR5 inhibition on colony formation ability were investigated by transfecting the cells with gene specific siRNA or exposure to maraviroc (IC_20_). Following 48 h of treatment in both cases, equal cell numbers were re-suspended in a semi liquid medium and allowed to form colonies for 6–8 days (large ≥ 30 cells, small < 30 cells) (**a**). Trans-well chamber and scratch assays demonstrated that targeting CCR5 via siRNA or the antagonist (IC_20_) reduced the directional migration of CRC cells either towards a source of nutrients (FBS) or other cancer cells in vitro (**b**, **c**). Experiments were repeated at least twice and minimally three replicates to validate the results. Asterisks above the bars indicate statistically significant differences among control and treated groups (*P < 0.05, **P < 0.01, ***P < 0.001)

Migration from primary sites of origin and colonization of distant organs are crucial malignancy-related properties of cancer cells. CCR5-dependent migratory and clonogenic abilities of CRC cells were assessed in vitro by trans-well chamber migration, scratch healing and colony formation assays, respectively. Exposure to gene-specific siRNA or low concentrations of maraviroc (IC_20_) abrogated the colony formation and migration of CRC cells (Fig. 2). Overall, siRNA-mediated CCR5 inhibition was more effective in reducing colony formation of CRC cells than maraviroc-mediated blockage. The reason for this difference may be due to the duration of the initiating event, with other words, the inhibitory interaction of maraviroc with the receptor may have caused a shorter response than the reduced expression of the receptor, which needed re-synthesis for regaining function. As colony formation involves an observation period of about 1 week, small differences can sum up to significant variations. Additionally, the human cell lines were less responsive towards maraviroc exposure than the rat cells (CC531), especially when considering the ability to form large colonies (Fig. 2a). Migratory responses, either towards increasing concentrations of chemo attractants (FBS) in migration assay, or filling the scratched area (wound healing assay), demonstrated that CCR5 inhibition significantly hinders the directional migration of CRC cells (Fig. 2b, c). Considering the partial inhibition of migration due to the potential anti-proliferative activity in response to CCR5 knock down via siRNA, equal numbers of the cells were transferred to migratory chambers after the transfection period (48 h) to minimize these additive effects. In case of CCR5 blockage via maraviroc (IC_20_), as the cells were allowed to migrate in the presence of test compound, effects on migrations were normalized by percentage of survival. Overall, the data suggest significant abrogation of colony formation and migration of the CRC cells via CCR5 inhibition.

### CCR5 blockage modulates cell cycle-related signaling cascades in CRC cells

**Fig. 3:**
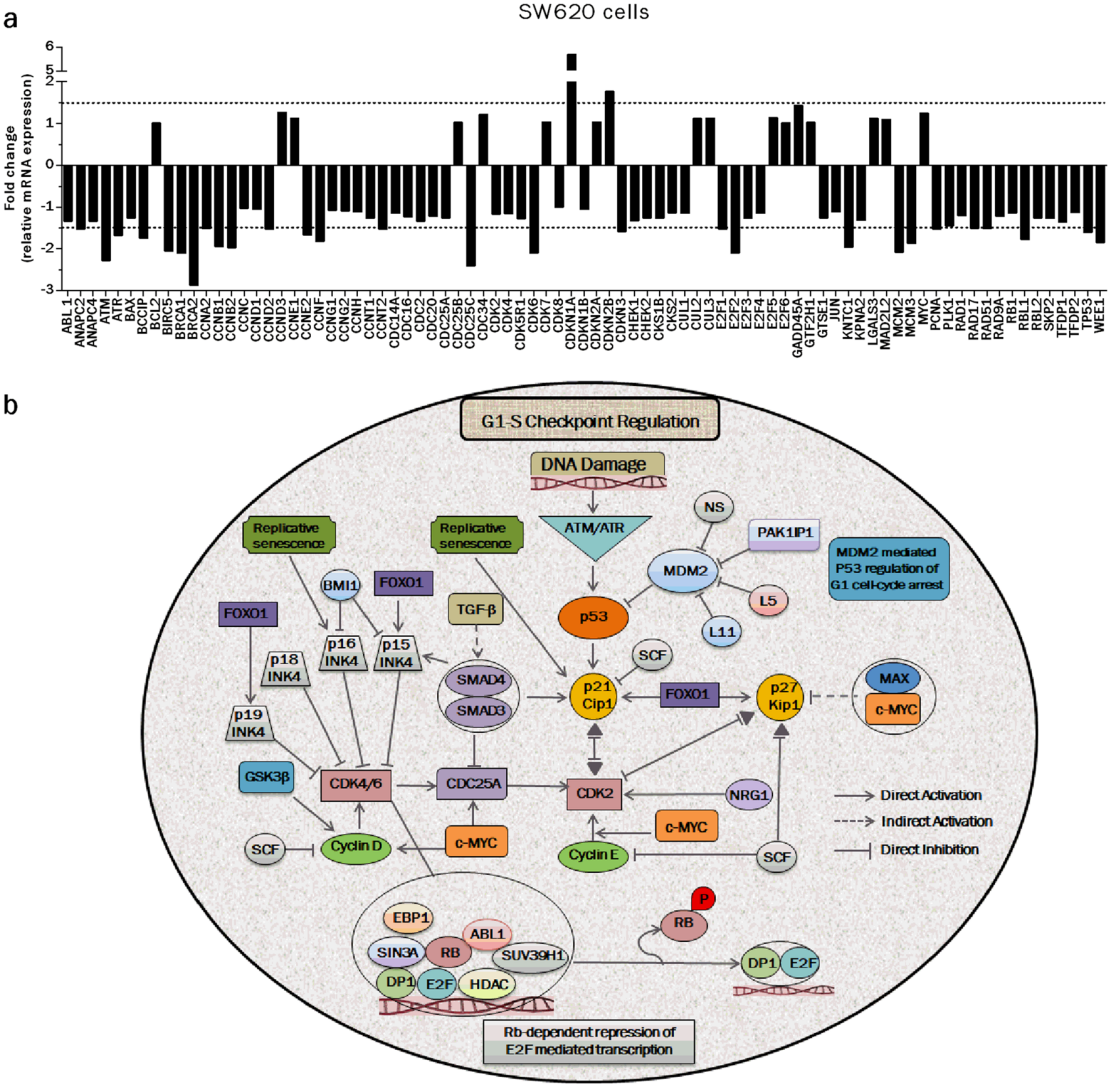
CCR5 blockage by maraviroc interferes with cell cycle-related signaling pathways. Human CRC cells (SW620) were exposed to maraviroc (IC_75_/48 h) followed by the expressional profiling of 84 cell cycle relevant genes by using a ready-made human cell cycle panel and qRT-PCR methodology. Following the normalization of control and experimental data sets, 2-ΔΔCt method was used to analyze relative expressional levels of the genes. Significant alterations in expression (≥ 1.5-fold, dotted lines) were observed in 35% of the genes (29/84) in response to maraviroc exposure (**a**). The expressional data set of the genes was used to draw a schematic signaling model of the cell cycle with the help of *Ingenuity Pathway Analysis*. The designed pathway revealed that CCR5 inhibition primarily interferes with the G1-S phase checkpoint regulation of the cell cycle in CRC cells (**b**)

Deregulation of cell cycle control is one of the basic hallmarks of cancer and often requires reprogramming of cell cycle-related cascades. In a previous study, we identified that CCR5 blockage by maraviroc induces arrest in the G0/G1 phase of the cell cycle (Pervaiz et al. 2015). The observed cytostatic effects had been more prominent in metastatic (SW620) than in primary (SW480) CRC cells. Based on these findings, SW620 cells were selected for elaborating the molecular reasoning underlying these previously observed effects. They were exposed to a relatively high concentration of the test compound (IC_75_/48 h) to monitor the potential alterations meticulously. Thereafter, expressional levels of the 84 cell cycle-related genes were determined by using a ready-made qRT-PCR-based panel. The panel included important players of the cell cycle like cyclins, CDKs, inhibitors, inducers and facilitators of the cell cycle. Blockage of CCR5 by maraviroc altered the expression (≥ 1.5-fold) of 29/84 genes (35%) of the panel in SW620 cells (Fig. 3a). Afterwards, based on the identified expressional profiling, a schematic model was proposed with the help of *Ingenuity Pathway Analysis* to describe CCR5-mediated alterations in cell cycle-related signaling cascades (Fig. 3b). This analysis highlighted “Cell Cycle: G1/S Checkpoint Regulation” and “Cyclins and Cell Cycle Regulation” as primarily involved canonical pathways in response to CCR5 blockage in CRC cells. At gene level, TP53 (a tumor suppressor), CDKN1A/P21 (cell cycle inhibitor) and transcription factors (FOXM1 and E2F1) were indicated as the major up-stream regulators of these canonical pathways.

### Activation of CCR5 axis is required for CRC liver metastasis

**Fig. 4:**
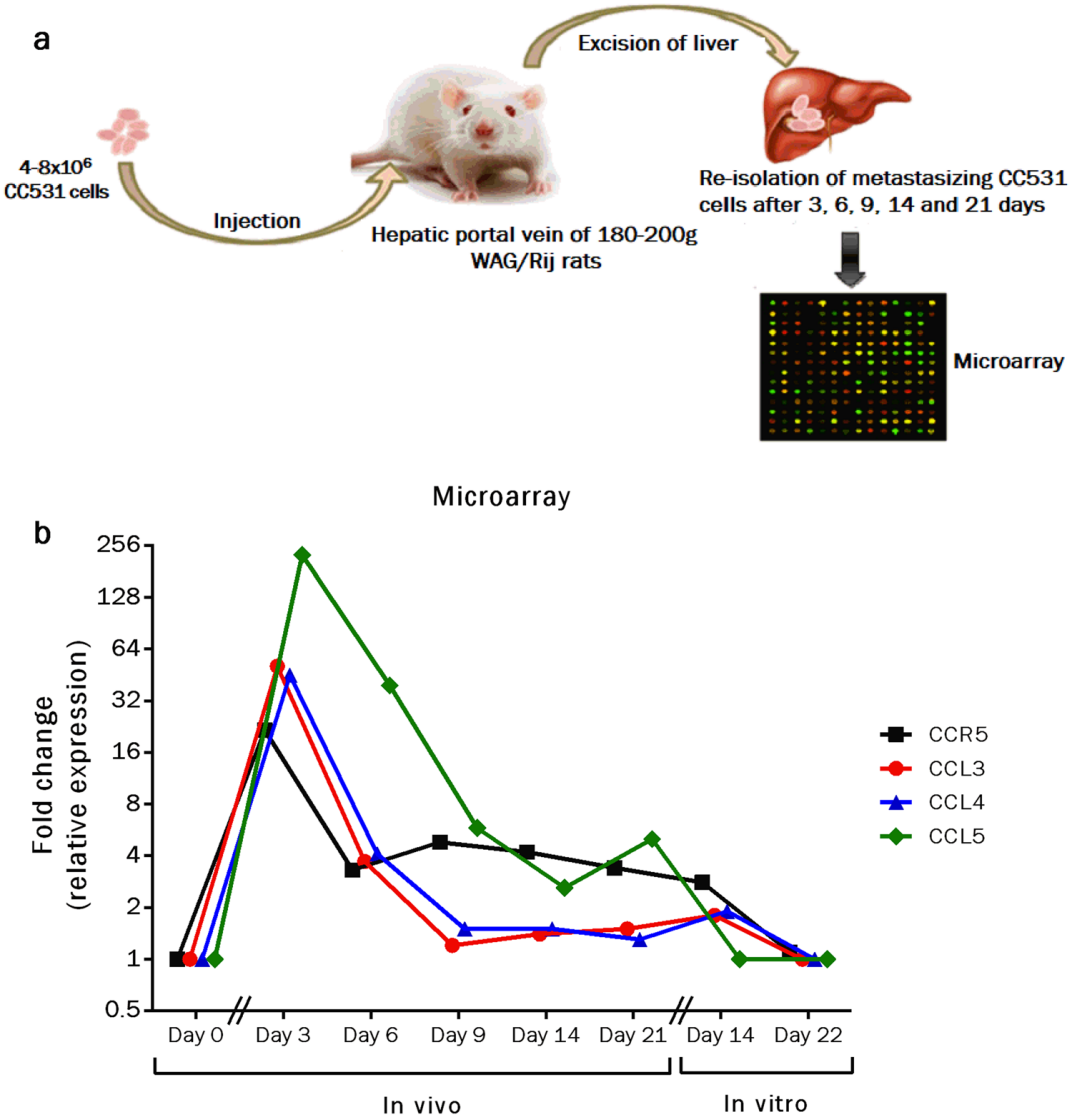
CCR5 axis up-regulation is observed in CRC liver metastasis. Rat CRC cells (CC531) were implanted into rat livers via the hepatic portal vein. Following the re-isolation of tumor cells after discrete time intervals (3, 6, 9, 14, 21 days), expression levels of the genes were identified by cDNA microarray methodology (**a**). The data revealed significant induction in expression levels of the CCR5 gene (22-fold) and its cognate ligands (CCL3: 51-fold, CCL4: 45-fold, CCL5: 225-fold) especially during the early time interval (3 days). The expressional levels almost came back to normal during the last time interval (21 days), when compared to re-isolated (after 21 days) CC531 CRC cells, which were cultured in vitro for 14 and 22 days (**b**)

Cancer cell metastases often require significant expressional modulations for successful survival in a given secondary organ. To evaluate the potential role of the CCR5 axis in CRC liver metastasis, rat CRC cells (CC531) were transplanted into rat livers via the hepatic portal vein. Following the re-isolation of tumor cells after discrete intervals (3, 6, 9, 14 and 21 days), expressional profiling was monitored by cDNA microarray methodology. The results were complemented by microarray data from cells which had been re-isolated at day 21 after implantation and then grown in vitro for 14 and 22 days. Overall, significantly increased expression of CCR5 and its cognate ligands (CCL3, CCL4, and CCL5) was observed in CC531 CRC cells at different stages of liver colonization (Fig. 4). Remarkably, the peak of this modulation was clearly at the earliest time interval following tumor cell implantation. The induction ranged from 22 to 225-fold. Clearly, there was an inverse relation between the degree of colonization and the elevation of expression levels. Additionally, among the three ligands of CCR5, CCL5 was consistently most altered for all selected intervals. To summarize, significant induction of the CCR5 axis is evident during CRC liver metastasis.

### CCR5 blockage suppresses CRC liver metastasis in an animal model

**Fig. 5:**
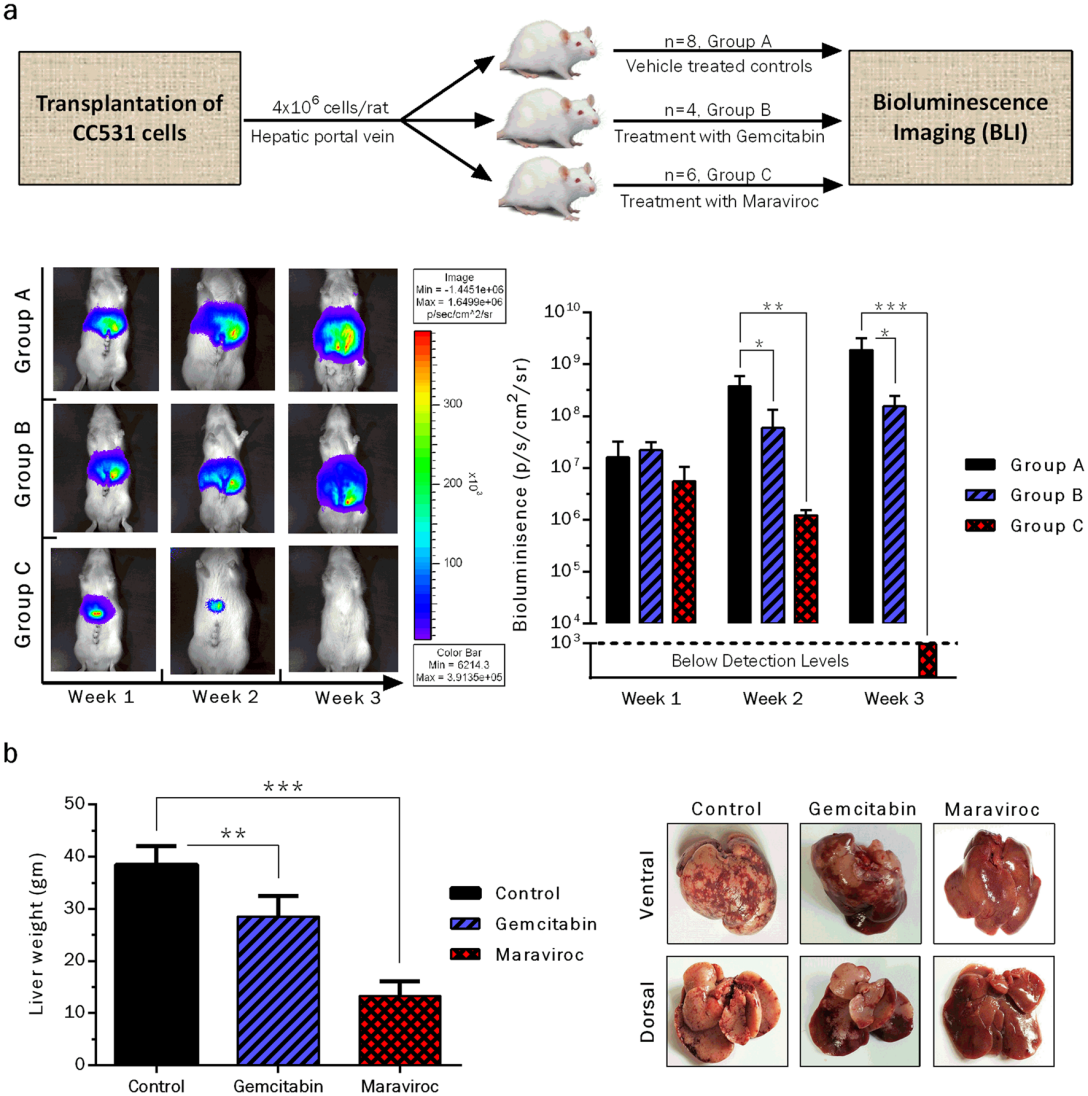
CCR5 inhibition with maraviroc inhibits CRC liver metastasis. Following implantation of CC531 cells into rat livers via their hepatic portal vein, the animals were treated by intra-peritoneal injections with gemcitabine (50 mg/kg/week), maraviroc (25 mg/kg/day) or vehicle only. Tumor growth and treatment responses were monitored via bioluminescence imaging for 3 subsequent weeks. A continuous tumor growth was observed in control animals, while there was moderate reduction in tumor burden after week 2 and 3 of treatment with gemcitabine. Treatment with maraviroc significantly reduced the tumor growth after 2 weeks and even induced complete remission in all rats of this group after 3 weeks (**a**). The rats were sacrificed at the end of experimental period and livers were excised and weighed. Distinctly higher liver weights were observed in control rats (± 40 g), while there was some reduction in gemcitabine-treated animals (± 30 g). In contrast, the livers of maraviroc treated rats were significantly lower in weight (± 12 g). Asterisks above the bars indicate statistically significant differences between control and treated groups (*P < 0.05, **P < 0.01, ***P < 0.001) (**b**)

A variety of in vitro antineoplastic effects observed after CCR5 inhibition as well as significant induction of the CCR5-axis during liver metastasis (cDNA microarray) compelled us to evaluate the efficacy of maraviroc in inhibiting CRC liver metastasis in an animal model. Following implantation of CC531 cells into the liver via the hepatic portal vein, rats were either treated with vehicle only (Group A, controls) or gemcitabine (Group B: 50 mg/Kg/week) or maraviroc (Group C: 25 mg/Kg/day). Gemcitabine was preferred over 5-fluorouracil, which is another well-known standard drug for treating CRC clinically, as the former had been found more active than the latter in the model used (Seelig et al. 2004). Tumor growth was measured by bioluminescence imaging (BLI) in all animals for 3 weeks following tumor implantation procedure (Fig. 5a). There was continuous tumor growth in control group A, while treatment with gemcitabine induced moderate tumor growth inhibition when compared to the vehicle-treated control animals. Treatment with maraviroc significantly inhibited the tumor growth as witnessed by complete remission of the tumor in 4/6 (66%) of the rats after week 2 following tumor implantation. The remaining animals of group C (2/6, 34%) were also found negative for any detectable luciferase signals after 3 weeks of treatment with maraviroc. At the end of this experiment, all rats were sacrificed and their livers were excised and weighed. Considering 10–12 g as normal liver weight for a 9–11-week-old male rat, a significant increase in mean liver weight was observed in the control (± 40 g) and gemcitabine groups (± 30 g). In contrast, liver weights of maraviroc-treated rats ranged around 12 g as shown in Fig. 5b. Taken together, these in vivo experiments demonstrated a significant potential of maraviroc to abrogate the growth of CRC cells in liver and to induce even complete remission of liver metastasis. Concomitantly, there were no signs of maraviroc-induced toxicity in the treated rats.

### Differential circulatory levels of CCR5 ligands in CRC patients

**Fig. 6:**
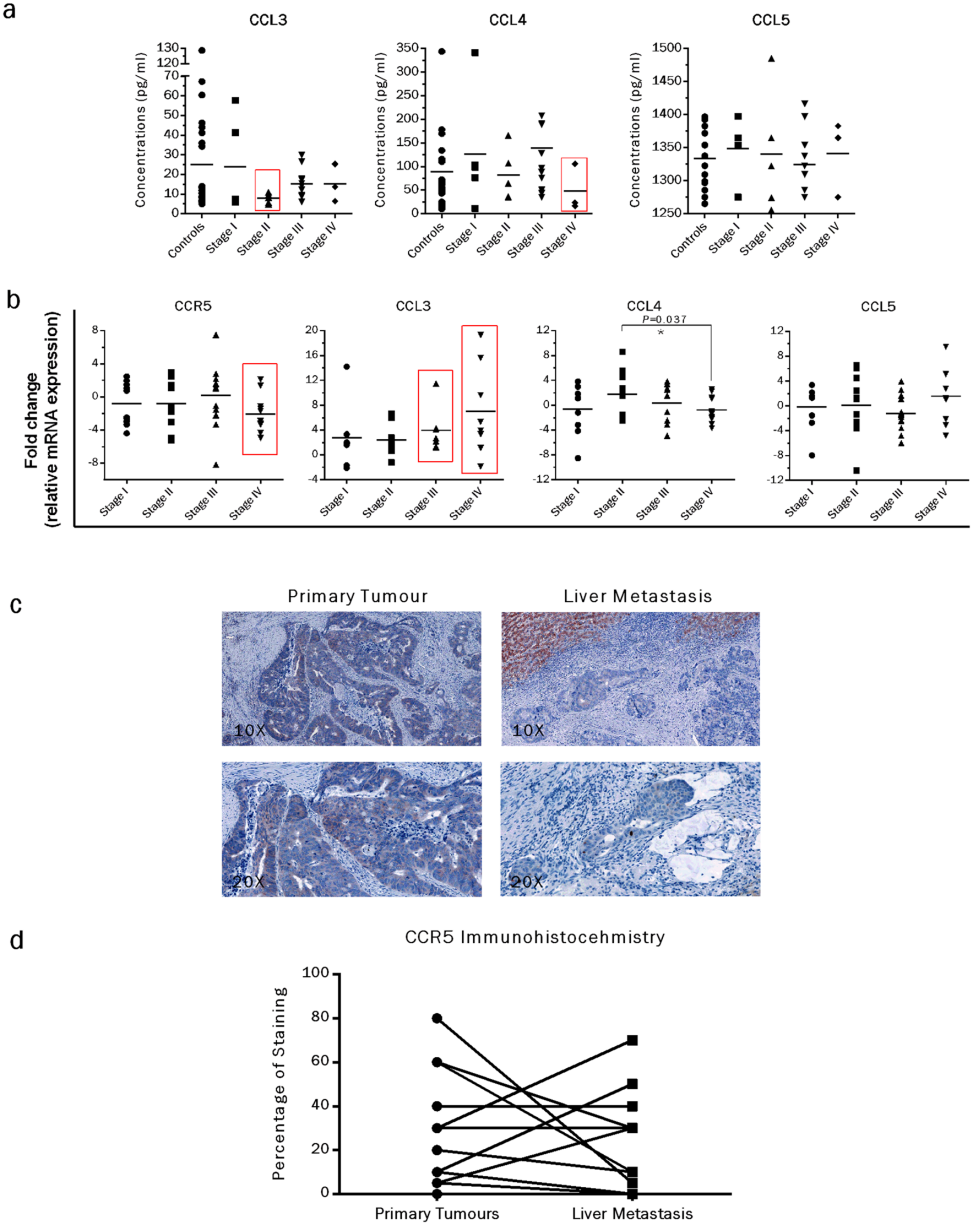
Circulatory and cellular expressional levels of CCR5 axis fluctuate among healthy controls and CRC patients. Circulatory levels of the three ligands of CCR5 receptor were determined by ELISA in serum samples from CRC patients and compared with healthy controls. Comparing the average concentrations of healthy controls, CCL3 was the ligand with the lowest values (27 pg/ml) as compared to CCL4 (92 pg/ml) and CCL5 (1335 pg/ml). When compared with healthy controls, considerable reduction of CCL3 and CCL4 was observed in stage II and stage IV CRC patients, respectively (see boxed values). In variance, CCL5 levels showed no significant differences as compared with healthy controls (**a**). Tumor associated transcriptomic profiling of CCR5 gene and its related ligands was identified via qRT-PCR methodology. When compared with healthy mucosa specimens, a reasonable reduction in expression (− 2.1-fold) of the CCR5 gene was observed only in stage IV CRC patients. In contrast, a gradual increase in CCL3 expression was observed in patients with late stages of CRC (stage III: 3.9-fold, stage IV: sevenfold). In case of CCL4 and CCL5, expression levels were not altered significantly (< twofold) in CRC patients as compared to healthy controls (**b**). Histopathological expression levels of CCR5 were identified by immunohistochemistry in 15 CRC and matched liver specimens. When compared, isolated positive cells (< 10% positive cells) were more frequent in primary tumors (5/15, 33%) than in liver metastasis samples (3/15, 20%). In contrast, focally positive cells (10–70% positive cells) were more prevalent in liver metastasis specimen (8/15, 53%) as compared to primary tumors (6/15, 40%) (**c**, **d**)

The potential significance of our experimental data compelled us to expand the work to patient samples. As a first step, circulatory levels of three cognate ligands of the CCR5 receptor (CCL3, CCL4, CCL5) were determined in serum samples of CRC patients and compared to an equal number of healthy controls. Demographic data about all the enrolled patients and healthy controls are shown in Table 2. All three ligands showed a relatively large range of values as determined by a specific ELISA (Fig. 6a). When comparing the mean values, CCL3 circulatory levels were almost similar in controls (27 pg/ml) and stage I patients (24 pg/ml), while there was a marked reduction in stage II patients (8 pg/ml) followed by a normalization in stage III and IV patients (15 pg/ml for each). In case of CCL4 circulatory levels, there was some induction in stage I (1.4-fold, mean 126 pg/ml) and stage III patients (1.5-fold, 140 pg/ml) as compared to healthy controls (92 pg/ml). Interestingly, CCL4 levels dropped noticeably in stage IV patients (1.9-fold, 49 pg/ml) and remained almost constant in stage II (81 pg/ml) when compared with the controls. There were marginal differences in circulatory levels of CCL5 in healthy controls and CRC patients. Nevertheless, CCL5 was the most abundant CCR5-related ligand in our clinical samples (1324–1349 pg/ml) followed by CCL4 (49–140 pg/ml) and CCL3 (8–27 pg/ml). Overall, the data suggest stage-dependent variations of at least two cognate ligands (CCL3, CCL4) of the CCR5 receptor, when comparing the average circulatory levels with healthy controls.

**Table 2:** Demographic data of clinical samples

Parameter	Lahore patients	Nürnberg patients	Heidelberg patients
Analysis	ELISA	Real-time PCR	IHC
Number of patients	24	51	15
Number of healthy controls	24	10	N/A
Age (average) years	42.3	65.8	64.3
Gender	11 male/13 female	33 male/ 18 female	9 male/ 6 female
Location	12 colon/ 12 rectum	35 colon/ 16 rectum	6 colon/ 9 rectum
Tumor stage (UICC)	I (5)	I (10)	I (1)
	II (5)	II (15)	II (12)
	III (11)	III (15)	III (2)
	IV (3)	IV (11)	–

### Transcriptomic expressional patterns of CCR5 axis vary in CRC patients

To assess the expressional profile of CCR5 and its cognate ligands at transcriptomic levels, we analyzed 51 surgically resected primary human CRC tissues and 10 healthy mucosa samples by qRT-PCR methodology (Fig. 6b). Overall, there were no statistically distinct changes in CCR5 expression of CRC patients. However, a noticeable inhibition of CCR5 expression (− 2.1-fold mean) was observed in stage IV patients when compared with the healthy specimens. For CCL3, almost a gradual increase in expression levels was noticed for late stage CRC, as evident by 2.7, 2.4, 3.9 and 7.0-fold increased levels for stage I, II, III and IV, respectively. Expressional patterns of CCL4 were not different from that of controls except for stage II CRC patients (+ 1.7-fold induction). CCL5 expression was least altered in CRC patients when calculated averages were compared with that of healthy controls. To summarize, CCR5 and its cognate ligands (CCL3, CCL4, CCL5) showed stage dependently varied expressional profiles at transcriptomic levels when compared with the appropriate controls.

### Histopathological profile of CCR5 in primary and metastatic CRC patients

To determine the CCR5 expression at protein level, we analyzed 15 primary tumors and correspondingly matched liver metastasis specimens of CRC patients by immunohistochemistry (Fig. 6c, d, Table 3). Among the selected pool, 12/15 (80%) of the patients were classified as grade 2 primary tumors. Among these, following the CCR5 staining, 5/12 (42%) were found either negative or with isolated positive cells (< 10% positive cells), while 7/12 (58%) were focally positive (10–70% positive cells). When compared with the matched liver metastasis specimens, isolated positive cells were more prevalent in primary tumors (5/15, 33%) as compared to liver metastasis samples (3/15, 20%). In contrast, focally positive cells were predominant in liver metastasis tissues (8/15, 53%) in comparison to primary tumors (6/15, 40%). The data suggest a relatively higher CCR5 expression burden in CRC liver metastasis as compared to the primary tumor tissues.

**Table 3:** Immunohistochemical staining of CRC samples and corresponding liver metastases

Sr. No	TNM Classification	^(a/b)^CCR5		^(c)^Liver metastases occurrence
		Primary tumor	Liver metastasis	
1	pT3 pN1b (3/26), G2	5%, i1	30%, i1-2	M
2	pT3 pN2b (10/19), G2	30%, i1-2	30%, i1	M
3	pT4a pN2a (5/22), G3	60%, i1-2	10%, i1	S
4	pT3 pN0 (0/11), G2	40%, i1	40%, i1	S
5	pT3 pN0 (0/17), G2	10%, i1-2	0	S
6	pT3 pN0 (0/21), G2	30%, i1-2	30%, i1	M
7	ypT3m ypN2a (5/14), G(2)*	30%, i1-2	70%, i1-2	S
8	pT3 pN1b (2/8), G2	0	0	M
9	pT3 pN2b (12/18), G2	5%, i1	30%, i1-2	M
10	pT3 pN0 (0/20), G1	5%, i1-2	0	M
11	pT4a pN2b (10/15), G2	0	0	S
12	ypT3 ypN2a (4/18), G(2)*	20%, i1-2	10%, i1	M
13	pT3 pN0 (0/15), G2	80%, i2-3	5%, i1	M
14	pT4a pN0 (0/21), G3	10%, i1-2	50%, i1	M
15	ypT3 pN0, (0/10), G(2)*	60%, i1-2	30%, i1	M

^(a)^Staining intensity: (i) 0 negative, 1 weak, 2 moderate, 3 strong

^(b)^Frequency of CCR5 positive tumor cells: isolated positive cells: < 10%; focally positive: 10–70%; diffusely positive: > 70%

^(c)^M: metachronous, S: synchronous

*Grading may not be applied as of neoadjuvant treatment

## Discussion

Accumulating evidences have shown that CCR5 along with its ligands plays an important role in tumor progression and organ specific homing of cancer cells during metastasis. Based on these findings, strategies are being materialized for blocking the CCR5 axis to uncover resulting antineoplastic effects and therapeutic relevance in cancers (Aldinucci and Casagrande 2018; Casagrande et al. 2019; Mencarelli et al. 2013; Ochoa-Callejero et al. 2013; Suarez-Carmona et al. 2019; Tan et al. 2009; Velasco-Velazquez et al. 2012). Regarding CRC prognosis and its metastasis, the CCR5 axis has earned considerable attention over the last few years as a novel biomarker and therapeutic option (Cambien et al. 2011; Chen et al. 2019; Nishikawa et al. 2019; Zimmermann et al. 2010). We have also contributed to this notion recently and showed that targeting CCR5 by an FDA-approved antagonist (maraviroc) induces anti-cancer effects and inhibits the tumor growth in vivo (Huang et al. 2020; Pervaiz et al. 2015, 2019). In the present study, we validated that targeting the CCR5 receptor via RNAi or an antagonist induces significant antineoplastic effects, including inhibition of proliferation, migration, colony formation and interference with cell cycle-related signaling cascades. Furthermore, implantation of CRC cells in rat liver (mimicking a CRC liver metastasis model) revealed a course-dependent induction of the CCR5 axis during liver colonization. Targeting the CRC cells via maraviroc in this liver metastasis model led to complete remission of growing liver metastasis. Lastly, circulatory- and tumor-associated expression changes of genes related to CCR5 axis were assessed in primary and metastatic clinical CRC samples.

Maraviroc, a competitive (non-allosteric) antagonist of the CCR5 receptor, was originally designed as an entry inhibitor for R5-HIV infections. Owing to mounting importance of the CCR5 axis in cancer, maraviroc turned out to be an immediately available drug for therapeutic purposes (Blanco and Ochoa-Callejero 2016). Characterized by a favorable pharmacological profile and minimal liver toxicity, the compound has been used recently in a phase I clinical trial (NCT01736813) to treat patients with CRC liver metastasis (Halama et al. 2016). In this particular study, when patients with metastatic CRC were given maraviroc (300 mg twice per day), Halama et al. highlighted the pro-tumor effects of infiltrating immune cells via the CCR5 axis. Interestingly, blockage of CCR5 by maraviroc re-polarized the immune cells to cause anti-tumor effects and reduced the subsequent disease burden. Profound success of this first clinical trial has attracted considerable attention of the scientific and medical community to further explore the CCR5 axis for treatment of advanced stage CRC. Currently, another phase I clinical trial (NCT03274804) is going on, where patients with refractory microsatellite stable metastatic CRC are being treated with a combination of pembrolizumab (anti-PD-1 antibody) and maraviroc. This trial possibly will reveal a new horizon in using a CCR5 antagonist like maraviroc in combination with other targeted agents. As these clinical studies have highlighted the concept of immune remodeling via the CCR5 axis, our pre-clinical data point to a direct anticancer effect of maraviroc in breast, pancreatic and CRC cells (Huang et al. 2020; Pervaiz et al. 2015, 2019). Furthermore, the concentrations used in our in vivo studies (25 mg/kg) are in the pharmacological range as shown by a calculated human equivalent dose of 242 mg/day (Nair and Jacob 2016). Based on these studies, it can be hypothesized that targeting the CCR5 axis implies using a double edged sword with direct antineoplastic effects against tumor cells and remodeling the immune system for ensuing anti-tumor effects.

In the present study, we identified significant anti-proliferative effects by targeting CCR5 via either gene specific siRNAs or maraviroc. Interestingly, siRNA-mediated knockdown led to a pronounced inhibition of CCR5 at mRNA (60–80% after 48–72 h) but not at protein levels (< 40%). The reduced inhibition at protein level could be due to the long half-life of the CCR5 protein present in membrane structures or to epigenetic cellular feedback loop(s) to maintain certain CCR5 protein levels. In spite of the poorly affected protein levels, targeting CCR5 inhibited the survival of selected human (SW480: primary, SW620: metastatic) and rat (CC531) CRC cell lines in vitro. A possible explanation is that CCR5 interacts with multiple ligands and various signalling cascades to play a pivotal role in metabolic and proliferative events (Gao et al. 2017; Oppermann 2004). Thus, it is not too surprising to witness a substantial inhibition of cell survival even after a small change in protein levels following siRNA knockdown. As far as maraviroc is concerned, relatively high concentrations (1.5–750 µM) were used in the in vitro part of this study. However, when tested clinically, the test compound was well tolerated in healthy persons (up to 1200 mg/day) and patients with viral infections and cancers (up to 300 mg/day twice daily) with no clear adverse effects on haematology and hepatobiology (Emmelkamp and Rockstroh 2007; Halama et al. 2016). Given the aggressive and invasive nature of CRC cells, we evaluated the importance of the CCR5 axis for cellular invasion and metastasis. Inhibition of CCR5 led to a decline in cell movement, invasiveness and colony formation ability (Mencarelli et al. 2013; Pervaiz et al. 2019; Singh et al. 2018; Velasco-Velazquez et al. 2012). Keeping in mind the primary chemo-attractant property of any chemokine axis, the possible inhibition of migratory activities of cancer cells can be foreseen after inhibiting a vital axis like that of CCR5. In addition to migration, chemokines have been shown to affect important functional aspects like cellular proliferation, apoptosis and cell cycle (Legler and Thelen 2018). In a previous study, we observed a significant cell cycle arrest in G1 phase of the cell cycle in CRC cells after blocking CCR5 by maraviroc (Pervaiz et al. 2015). In the present study, we explored potential signaling cascades underlying the previously observed cytostatic effects. Expressional profiling of 84 cell cycle-related genes followed by *Ingenuity Pathway analysis* (IPA) revealed that CCR5 primarily interferes with the “G1/S checkpoint regulation” in CRC cells. In the light of available reports and our data (Fig. 3), we envision that CCR5 blockage leads to the alteration of multiple genes and related pathways of the cell cycle. Nevertheless, investigations that are more detailed are required to understand the CCR5-mediated effects on cell cycle-related signaling cascades in depth.

As we know, liver metastasis is a lethal condition and accounts for almost more than 50% of CRC-related deaths. Cellular processes and complex underlying molecular events, responsible for CRC liver metastasis, are poorly understood. Thus, there is a pressing need to identify metastasis-related changes in the tumor cells. More importantly, it is required to relate molecular changes accurately to their time of occurrence, so that target genes and pathways could be manipulated at the right time for therapeutic purposes. To understand time-dependent metastasis-related genetic changes, CC531 cells were implanted in rat livers and re-isolated for expressional profiling by cDNA microarray. The analysis revealed significant induction of the CCR5 axis in CC531 cells during the initial phase (3 days) of liver colonization. Remarkably, at later stages this increase was less impressive and almost normalized at the final stage (21 days) of liver colonization (Fig. 4). Which factors imposed these dynamic alterations on the CCR5 axis is an open question that deserves more attention. Here, we can speculate about the influence of the tumor microenvironment playing a pivotal role during the progression of cancers. Specifically, interactions of the implanted CRC cells with liver cells and/or immunological effector cells could be driving forces in the transient changes of the CCR5 axis. Furthermore, the possibility of epigenetic modifications within the tumour cells cannot be ruled out, which may lead to marked induction of the CCR5 axis. From a clinical perspective, the transient early up-regulation of the CCR5 axis should be investigated following resection of a primary CRC for improving the treatment options by e.g. reducing the rise of CCR5 ligands in the liver environment. Present data show a significant role of the CCR5 axis during early liver metastasis and indicate a period during which the respective CRC cells are sensitive towards CCR5 blockade. Keeping in mind the multiple functions of the CCR5 axis, including cellular adhesions, proliferation, survival and immune modulation to support tumor growth in a secondary organ (liver), targeting the CCR5 axis at this period should have profound effects against metastasis development.

To validate our above-mentioned hypothesis, CC531 cells were implanted in rat livers followed by treatment with daily intra-peritoneal administration of maraviroc (25 mg/Kg/day). To assess the sensitivity of the tumor cells to a chemotherapeutic agent, a second animal group was treated with gemcitabine in parallel (50 mg/Kg/week). In untreated animals, a continuous growth of tumor cells was observed. Animals treated with gemcitabine showed a moderate reduction in tumor burden. Likely reasons for these marginal effects can be explained from the fact that gemcitabine is used clinically in combination with other drugs for maximum anticancer effects, while we used it as a single agent. Outstandingly, complete tumor remission (undetectable signals during BLI) was observed in animals treated with maraviroc during the in vivo experiments. Almost similar, but less impressive anticancer effects have been reported by others where significant reduction of growing tumor mass has been observed when using maraviroc in other malignancies (Casagrande et al. 2019; Mencarelli et al. 2013; Ochoa-Callejero et al. 2013; Pervaiz et al. 2019; Velasco-Velazquez et al. 2012). At the end of experiments, the pathology of rats, especially the liver weights were in line with the BLI data. Needless to say, that our in vivo experiments indicate that targeting the CCR5 axis using maraviroc is a highly promising therapeutic option for CRC liver metastasis.

Cancer-related activation or inhibition of a chemokine network is a well-known phenomenon. It allows the tumor cells to cross-talk with surrounding stromal/immune cells for dictating the further progression. Considering this, it is worth to investigate alterations in chemokine expression during various stages of a cancer (Bian et al. 2019; Borsig et al. 2014; Huang et al. 2018). A number of studies have shown differential expression of CCR5-related ligands (CCL3, CCL4, CCL5) in peripheral blood and tumor samples of CRC. Furthermore, these variations were associated with varied prognosis and treatment outcomes (Fuente et al. 2018; Halama et al. 2016; Nishikawa et al. 2019; Yamaguchi et al. 2019). In this study, we analyzed the circulatory and tumor-associated levels of CCR5 and/or its ligands (CCL3, CCl4, CCL5) via ELISA and qRT-PCR/IHC, respectively. The results supported our working hypothesis; circulatory levels of the CCR5 cognate ligands (CCL3, CCL4, and CCL5) differ in CRC patients, when compared to healthy controls. Differential expression of these ligands could play a vital role in overall CRC prognosis as they can mediate a crosstalk between tumor cells and surrounding microenvironment to promote further tumor growth at primary locations and/or metastatic niches. Additionally, varied expression of these chemokine ligands can be exploited as biomarkers to detect CRC. However, careful consideration should be given to the fact that circulating levels of the ligands may not represent the actual levels at the tumor sites. Therefore, our results related to circulatory levels of the CCR5 ligands in CRC patients should be validated on larger sample pools and other populations as well. As far as the tumor-associated expressional profile of the CCR5 axis is concerned, the majority of available data indicate induction of this chemokine network with a pro-tumor role and shorter overall survival rate in CRC (Cambien et al. 2011; Erreni et al. 2009; Nishikawa et al. 2019; Zhang et al. 2018; Zimmermann et al. 2010). Furthermore, a distinct pattern of CCR5 expression has been reported recently in metastatic CRC liver specimens. The authors showed that intensity of CCR5 expression increases with primary tumor size, while a “patchy” pattern of the receptor (at least 10% of tumor cells negative for the CCR5 in a patchwork-like configuration) was observed in liver metastases (Suarez-Carmona et al. 2019). In our selected patient cohort, differential expression of CCR5 and its ligands (CCL3, CCL4, and CCL5) was observed in primary CRC tumors (Fig. 6b). To be precise, we identified a reduced average expression of CCR5 with an increasing primary tumor mass when compared with the healthy mucosa. Likewise, we observed variations in CCR5 stains during immunohistochemistry of the primary CRC tissues and matched metastatic lesions (Fig. 6c, d). These observations, at least in part, can be explained from our in vivo microarray data, which clearly shows temporal induction of the CCR5 axis during early tumor growth in the liver. This phenomenon can be exploited from the therapeutic perspective as well, where CCR5 blockage can lead to abrogation of vital signaling cascades required for tumor growth during metastasis. However, further studies will be required to dissect and understand the precise contribution of the CCR5 axis in CRC progression, especially during metastasis.

To conclude, inhibition of CCR5 induces cytotoxic and cytostatic effects in CRC cells. In vivo data demonstrated significant induction of the CCR5 axis in CRC cells especially during the early phase of liver colonization. Likely, in a similar fashion of time-dependent expressional modifications, varied levels of CCR5 were observed in the clinical samples collected at various phases of patients with liver metastasis. Blocking the CCR5 receptor via maraviroc led to complete remission of the tumor in an animal model mimicking CRC liver metastasis. The findings highlight CCR5 as an attractive therapeutic target, where CRC patients with early-stage liver metastasis could be more responsive towards this treatment approach. In this context, maraviroc is an already available FDA approved CCR5 antagonist and can be used in clinical settings as a monotherapy or in combination with other agents to possibly cure patients having CRC liver metastasis.

## Electronic supplementary material

Below is the link to the electronic supplementary material.

Supplementary file1 (XLSX 12 kb)
